# A memristive plasticity model of voltage-based STDP suitable for recurrent bidirectional neural networks in the hippocampus

**DOI:** 10.1038/s41598-018-27616-6

**Published:** 2018-06-19

**Authors:** Nick Diederich, Thorsten Bartsch, Hermann Kohlstedt, Martin Ziegler

**Affiliations:** 1 Nanoelektronik, Technische Fakultät, Christian-Albrechts-Universität zu Kiel, D-24143 Kiel, Germany; 2 Department of Neurology, Memory Disorders and Plasticity Group, University Hospital Schleswig-Holstein, Kiel, Germany

## Abstract

Memristive systems have gained considerable attention in the field of neuromorphic engineering, because they allow the emulation of synaptic functionality in solid state nano-physical systems. In this study, we show that memristive behavior provides a broad working framework for the phenomenological modelling of cellular synaptic mechanisms. In particular, we seek to understand how close a memristive system can account for the biological realism. The basic characteristics of memristive systems, i.e. voltage and memory behavior, are used to derive a voltage-based plasticity rule. We show that this model is suitable to account for a variety of electrophysiology plasticity data. Furthermore, we incorporate the plasticity model into an all-to-all connecting network scheme. Motivated by the auto-associative CA3 network of the hippocampus, we show that the implemented network allows the discrimination and processing of mnemonic pattern information, i.e. the formation of functional bidirectional connections resulting in the formation of local receptive fields. Since the presented plasticity model can be applied to real memristive devices as well, the presented theoretical framework can support both, the design of appropriate memristive devices for neuromorphic computing and the development of complex neuromorphic networks, which account for the specific advantage of memristive devices.

## Introduction

Synaptic plasticity in the excitability between neurons results from an increase or reduction of the strength of synaptic connections and thus contributes to neuroplasticity. Neuroplasticity is the ability to adapt to and reorganize the structure or function to internal or external stimuli and occurs at the cellular, population, network or behavioral level. Therefore, it is reflected in the cytological and network architecture, as well as in intrinsic properties of neurons and circuits. Synaptic plasticity can be mathematically described within the framework of the Hebbian learning theory^1^. In this respect, Hebbian models allow the description of long-term potentiation (LTP) and long-term depression (LTD) and account for temporal coding schemes, such as spike-time-dependent-plasticity (STDP)^2–4^.

A variety of plasticity models have been developed which account for different temporal and functional aspects observed in electrophysiological investigations (for a review the reader is referred to refs^5,6^). Of particular interest are voltage-based STDP models^5–8^ in which synaptic changes are depending on pre-synaptic spike arrival and post-synaptic membrane potential. These models sufficiently describe the voltage and frequency dependence sufficiently, as well as several non-linear effects that are observed in electrophysiological investigations of neuroplasticity. A further advantage is that voltage-based STDP models belong to the class of phenomenological models which aim to provide a minimal description of the principal phenomena observed in electrophysiological investigations. Such an approach has the advantage of reducing the complexity of the system to a few key parameters and allows testing the compatibility of model parameters with experimental synaptic plasticity data (an overview of model classification can find in ref.^9^).

Besides purely mathematical plasticity models, the development of electronic circuits, so-called neuromorphic circuits, enables a physical implementation of learning models in hardware and might therewith overcome current restriction of serial, binary information processing in neuroinformatics^9^. Neuromorphic systems -invented by Carver Mead in the 80’s of the last century- employs analogue VLSI (Very Large Scale Integration) which is based on Si CMOS (Silicon Complementary Metal Oxide Semiconductor) technology^10,11^. Recently, this field gained new momentum with the advent of memristive devices. We consider a memristive system as a two-terminal electrical device which is able to remember the history of applied electrical stimuli^12–14^. In the last couple of years, temporal as well as frequency coding schemes have been successfully emulated with memristive systems^15–19^. In this context, a variety of different memristive devices, which based-on different physical concepts and materials, were employed to emulate STDP^15–19^. Thus, memristive devices provide an interesting innovative approach in emulating synaptic plasticity with the advantage of real-time applications in neuromorphic systems^15,16^.

In this study, we show that the use of memristive behavior provides an excellent framework for phenomenological modelling of cellular synaptic mechanisms. Features of cellular synaptic plasticity as well as network learning mechanisms are simulated by the characteristic of a memristive device. In detail, the memory behavior, the voltage- and history-dependence of memristive systems, is used to incorporate a voltage-based plasticity rule suitable to account for a variety of experimental plasticity data. The key features of the developed model are a memristive synaptic learning rule, which is determined by a non-linear and state dependent synaptic weight change according to^19^, as well as a voltage-based STDP inspired by^5–8^. Furthermore, using models of neuronal all-to-all connecting networks and neurobiological hippocampal circuits, we extend our findings of synaptic plasticity from the cellular to the network level. At this respect, principles of the discrimination and processing of mnemonic pattern information in the auto-associative CA3 network of the hippocampus are emulated and discussed. In detail, based on biological data we show evidence that our network model is capable of learning by the discrimination and completion of similar input patterns (pattern separation or completion) and by the formation of functional bidirectional connections resulting in the formation of local receptive fields in auto-associative CA3 networks.

## Modeling

### Synaptic plasticity model

**Figure 1:**
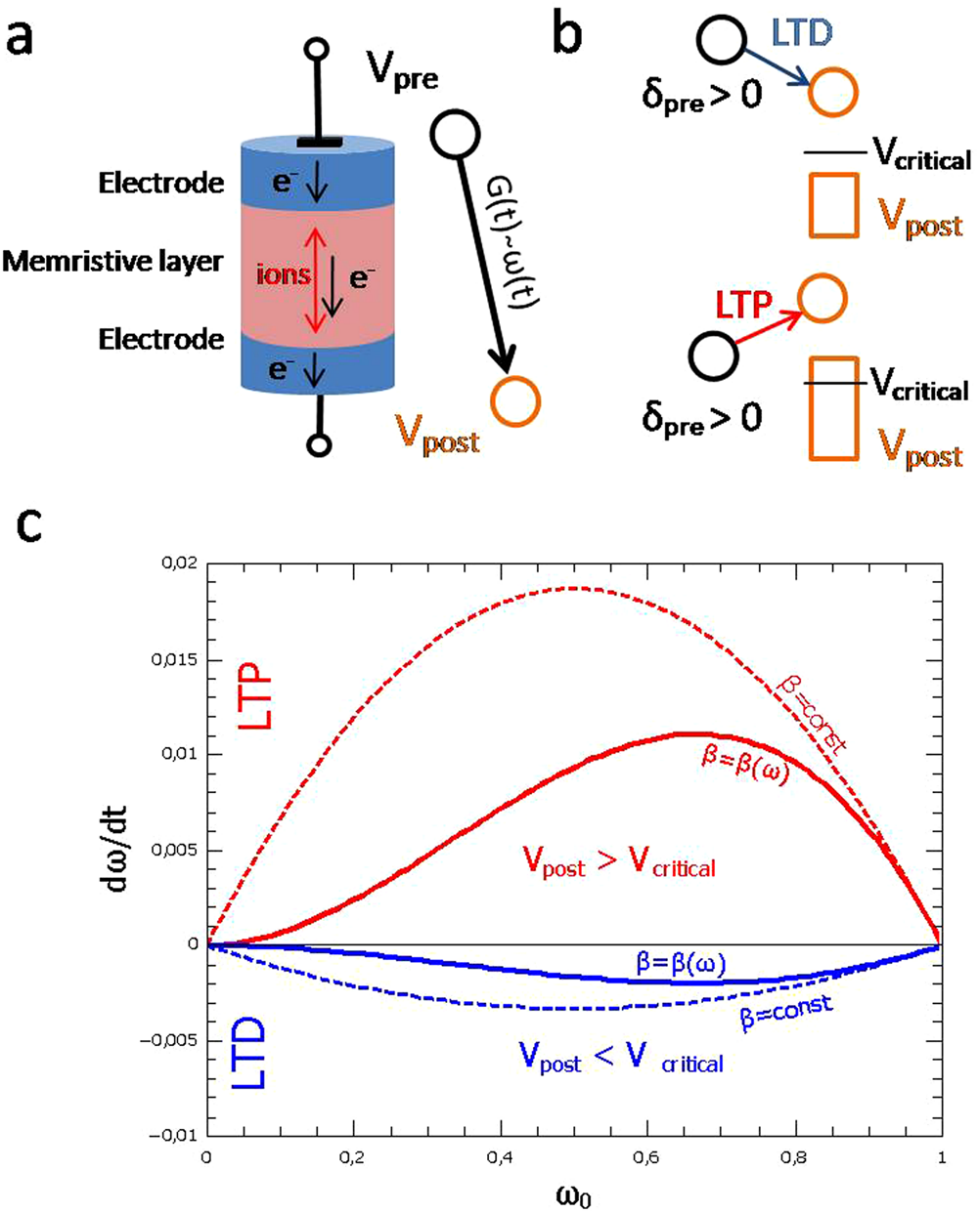
Memristive Plasticity Model: (**a**) Schematic drawing of an memristive device, which consists of a metal-insulator-metal (MIM) structure. A memristive device adapts his conductance to the previous applied electrical signals. Here, an oxide based memristive device in a voltage-controlled operation mode is sketched: the attributive memristive layer defines a solid-state-electrolyte in which ions are moved in dependence on the voltage and the voltage history. Regard to this unique behavior memristive systems are used for the emulation of synaptic functionality. In the memristive point of view the device conductance corresponds to the synaptic coupling strength, i.e. the synaptic weight variable *ω*. **(b)** Schematic drawing of the voltage potentials applied to the memristive device to increase or decrease the device conductance, i.e. the the synaptic weight *ω*(*t*): While the pre-synaptic potential *V*_*pre*_ is set to a constant value (for example the mass potential, i.e. zero volt) immediately after spiking, the post-synaptic potential *V*_*post*_ induces for values below a critical voltage *V*_*critical*_ long-term-depression (LTD) or above *V*_*critical*_ long-term-potentiation (LTP). Further, to account for the Hebbian learning procedure the device conductance is only affected when the pre-synaptic neuron is spiking (*δ*_*pre*_ > 0). **(c)** The memristive plasticity model provides a weight dependent learning process, i.e. the weight change dω/dt depends on the current value of ω_0_. LTP and LTD formation are shown (constant voltage pulses of 61.75 mV –lines colored red-_ and −11mV –lines colored blue- were used for LTP and LTD, respectively, while *V*_*critical*_ was set to zero.

The key content of the plasticity model is to use memristive functionalities, i.e. voltage and history dependence to model synaptic connectivity. Memristive systems can be realized as two terminal electronic devices, as sketched in Fig. 1(a). These devices adapt their resistance to the previously applied electrical stimuli and therefore, have a memory^16,17^. The memory derives from local changes in the atomic structure, which occurs within the so-called memristive layer (see Fig. 1(a)). This is in particular important for the realization of electronic artificial neural networks, so-called neuromorphic circuits^15,19,20^. However, although the structure of a memristive device is rather simple, the underlying functional principle are quite divers and a large variety of material systems have been realized so far^15,21–23^. In particular, this leads to a large variability in the current-voltage characteristics of the different memristive systems. In this context, it is helpful to use a generic device model, which is able to describe the characteristics of different memristive devices and which at the same time enables the emulation of synaptic functionality quite realistically. We apply a recently developed memristive model, that allows the description of common plasticity measurements of memristive devices^19^.

In synapses, conductivity is described by the concentration of neurotransmitters within the synaptic cleft, which depends on the activity of the pre-synaptic neuron and the number of activated post-synaptic ion channels^24^. Mathematically, this can be described by an ion channel-dependent conductivity^25^ *g*_*syn*_(*x*_*ion*_(*t*)), where *x*_*ion*_(*t*) is a gating variable counting the time-dependent number of activated ion channels. Thus, the synaptic current is given by *I*_*syn*_(*t*) = *g*_*syn*_(*x*_*ion*_(*t*)) *u*(*t*), where *u* is the membrane potential of the post-neuron. In the memristive picture, the device conductance *G* (memductance) is a state-dependent function, where the state *x*(*t*) changes in dependence of the applied electrical stimuli over time, i.e. the applied voltage *V* or current *I*. For the case of a voltage driven memristive device this can be mathematically formulated as^12–14^

1 $$I=G(x(t))\cdot V\,{\rm{and}}\,\frac{{\rm{d}}x}{{\rm{d}}t}=f(t,x,V).$$

Here, *f* is a function which describes the dynamics of the resistance variation of a particular memristive cell under voltage application^17^. In an one-dimensional model, *x*(*t*) can be linked to the conductance by *G*(*x*(*t*)) = *G*_*on*_ *x*(*t*) + *G*_*off*_ (1 − *x*(*t*))^14^, where *G*_*on*,_ *G*_*off*_ are the boundary conductance values. In this model, if *G* is normalized to the boundaries *G*_*on*_ = *1* and *G*_*off*_ = 0, then the state variable *x*(*t*) of the memristive device is proportinal to the conductance *G*. In the memristive synapse model the gating variable *x*_*ion*_(*t*) defines the memristive state, where the weight of the synapse *ω* is dependent on *x*_*ion*_(*t*). Since *ω* is limited to the interval from *ω*_*min*_ = 0 to *ω*_*max*_ = 1, it can be considered as a normalized conductivity, which gives the strength of synaptic transmission. Thus, d*x*/d*t* (from Eq. 1) corresponds to d*ω*/d*t*.

To emulate synaptic plasticity with memristive cells, appropriate voltage pulses with different amplitudes must be applied to the two terminals of the memristive device -named in the following as “pre” and “post”, respectively (cf. Fig. 1). In order to simulate synaptic plasticity with memristive devices, the respective potentials at the two terminals of the device must be changed so that the device increases (potentiation) or decreases (depression) its conductance, depending on the activity of the respective neuron. This is usually done by a suitable voltage function for the pre-synaptic potential *V*_*pre*_ and post-synaptic potential *V*_*post*_, which leads to a suitable voltage *V* across the device^17,21^. In our model the post-synaptic potential *V*_*post*_ is used to change the conductance state of the memristive synapse, while the pre-synaptic potential *V*_*pre*_ is held constant. As illustrated in Fig. 1(b), this enables the emulation of synaptic connectivity: if *V*_*post*_ is higher than a critical set point voltage *V*_*critical*_ the conductance of the memristive cell increases. While for the opposite condition, i.e. *V*_*post*_ < *V*_*critical*_, a decrease in the synaptic connection is evoked. Inspired by the models of refs^5–8^, we assume that the synaptic weight can be affected only in the case that the pre-synaptic neuron is active, i.e. spiking. With regard to this idea, synaptic spike trains can be mathematically formalized by a series of delta pulses *x*(*t*) = *Σ*_*k*_ *δ*(*t* − *t*^*k*^), where *t*^*k*^ is the *k*-th spike time of the pre-synaptic neuron. Hence, whenever the pre-neuron is spiking (*t* = *t*^*k*^) the synaptic weight *ω*(*t*) is changed. By combining this concept with that of a memristive system the weight updating process can be described by a function which depends on the actual weight *ω*(*t*) and the current post-synaptic potential *V*_*post*_:

2 $$\frac{d\omega }{dt}=\{\begin{array}{lll}f(t,\omega ,{V}_{post}) & -\,\kappa \omega (t), & {\rm{for}}\,t={t}^{k}\\  & -\kappa \omega (t), & {\rm{else}}\end{array}$$

Here, *f* describes the dynamics of the synaptic changes and has to be determined in relation to biological boundary conditions that define the memory capacity of the memristive synapse. $\kappa \omega (t)$ describes a constant leakage term ($\kappa  > 0$),which on the one hand describes a continuous depression observed in physiological nerve cells and on the other hand takes the retention characteristics of real memristive devices.

Three synaptic properties are of particular importance in this context^25^: locality, cooperativeness, and associativity. Locality, or in other words input specificity, means that the change of synaptic efficacy is critically dependent on the activity of two interconnected neurons by a specific synapse, but not on the activity of other neurons in the network. Cooperativeness means that the probability of the induction of LTP increases with the number of stimulated pre-synaptic neurons. Associativity bridges the gap of the one-dimensional cellular learning mechanism to the multi-dimensional network level. So that the system does not run into stable points of intrinsic given synaptic weight boundaries (*ω*_*min*_ < *ω*(*t*) < *ω*_*max*_ where *ω* ϵ {0, 1}), on the one hand we use *sup*(*ω*) = 0.05 and on the other hand a continuous forgetting rate. The *supremum* condition protects the system from losing a possible connection for all times, whereas the continuous forgetting rate is a protection against a steady maximized connection. These conditions reflect the anticipated behaviour of memristive devices, too. Following^19^, those requirements can be met by using a modified logistic differential equation for *f* in Eq. 1:

3 $$f(t,\omega ,{V}_{post})=\beta (t,\omega ,{V}_{post})\,\omega (t)\,(1-\frac{1}{{\omega }_{max}}\omega (t))$$

Here, *β* is the synaptic learning rate which is assumed to be weight and voltage dependent. Recently, we showed evidence that an appropriate choice of *β* allows the description of different memristive devices characteristics within the framework of this plasticity model^19^. For this investigation, however, we focus on synaptic plasticity measurements and extent our former model to a more neurobiologically relevant framework: we would like to address the question of how far the plasticity model accounts for experimental electrophysiological data. Therefore, we have chosen the following linear dependency for the synaptic learning rate *β*:

4 $$\beta (t,\omega ,{V}_{post})=k\,\omega (t)\,{V}_{post},$$

where *k* is a positive constant. Furthermore, to account for both depression (a down regulation of *ω*) and potentiation (up regulation of *ω*), a bipolar voltage is assumed for *V*_*post*_. Hence, either potentiation is induced, if *V*_*post*_ > 0, or depression occurs in the case that *V*_*post*_ is negative, as shown in Fig. 1(c). Therein, the adaptive behaviour of the plasticity model is presented, wherefore the weight change d*ω/*d*t* is plotted as function of the weight *ω*(*t*) itself, as highly possible for LTD and LTP with the used simulation parameter. For this simulation the maximal and minimal voltage for *V*_*post*_ has been used, respectively. In particular, it shows that for low and high values of *ω*(*t*) their changes convert to zero, which binds the weight, avoids an uncontrolled synaptic growth and therefore emulates an important characteristics of biological networks. Thus, ω_min_ and ω_max_ are stable points of the modified logistic differential equation for the decreasing or increasing case, respectively. Most sensitive for a weight modification is the model if the weights have middle values, i.e. between 0.4 and 0.8. This in particular ensures a bimodal weight distribution, as needed for asymmetric Hebbian learning rules, such as spike-timing dependent plasticity (STDP, see^26^) and differs therewith from a constant learning rate as shown in Fig. 1(c) (dashed lines).

### Neuron model

**Figure 2:**
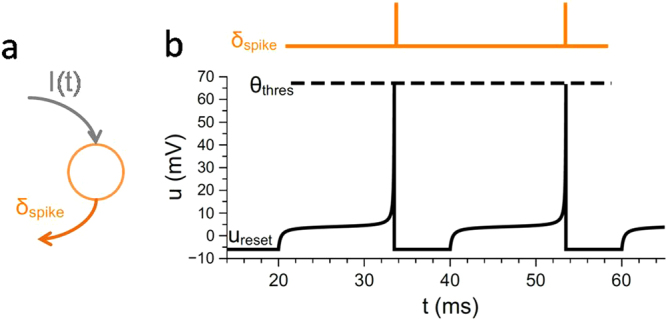
Quadratic-Integrate- and fire neuron model: (**a**) Schematic of the spiking neuron model in which a particular current input *I*(*t*) evoke a corresponding spike-pattern *δ*_*spike*_. (**b**) Obtained membrane potential *u*(*t*) for a constant input current: when the membrane potential reaches the threshold voltage *ϴ*_*thres*_ a spike is created. For the neuron model a quadratic-integrate-and fire model has been used. The used simulation parameters are shown at Table 1.

In the here proposed memristive plasticity model, the voltage course of *V*_*post*_ plays a crucial role. In particular, the polarity decides if the synaptic connection is potentiated or depressed, while the actual value of *V*_*post*_ together with the actual synaptic weight determines the amount of the synaptic change. Hence, a neuron model is required to describe the membrane potential evolution. A straightforward description of a neuron is given by the leaky-integrate-and fire model (LIF, see^1^): a membrane capacitance *C* is connected in parallel to a leaky conductor *g*_*L*_ and they are driven by an input current *I*(*t*), which simulate the ionic current through a synapse. With respect to the non-linearity and singularity of action potentials, the so-called quadratic-integrate and fire model offers a mathematically simple extension to LIF. This model can be expressed by.

5 $$C\cdot \frac{du}{dt}=-\,{\tilde{g}}_{L}\cdot ({u}_{critical}-u)({u}_{rest}-u)+I(t)$$

where ${\tilde{g}}_{L}$ has the dimension of conductance per voltage. Further, *u(t)* refers to the membrane potential of the neuron, while *u*_*rest*_ is its resting potential, which has to be set to an appropriate value in order to account for the bipolarity of the memristive plasticity model. Further, ucritical refers to the threshold potential for self-induced spiking. A sketch of the neuron model together with the representation of the membrane potential at constant input current is shown in Fig. 2, while the used parameters are summarized in Table 1. These parameters are selected as adaptation parameters for neuro-physiological data, the adjustment is explained in the first part of chapter ‘results’ and is shown in Fig. 2. In detail, the application of an input current evokes an integration of *u*(*t*) according to Eq. 5. If *u*(*t*) reaches the threshold potential *ϴ*_*thres*_, a spike is generated and the membrane potential is reset to *u*_*rest*_. Such a spike propagates to the downstreaming neurons in the network as a fixed current delta pulse. Further, to prevent the neuron from spiking directly after the depolarisation, a refectory period *t*_*ref*_ has been added. During its term *u*(*t*) is hold at *u*_*rest*_.

**Table 1:** Parameter for the plasticity and neuron model of the present simulation model, adapted to neuro physiological data.

Parameter	Value
θ_thres_, spiking threshold	61.75 mV
u_critical_, critical potential	9.00 mV
u_rest_, resting potential	−11.0 mV
C, membrane capacitance	1 mF
${\tilde{g}}_{{\rm{L}}}$, leak conductance	1 (ΩV)^−1^
V_critical_, set point voltage	0 V
k, learning rate	1.21 (ms)^−1^
κ, forgetting rate	4.17 10^−3^ (ms)^−1^
ω_min_, minimal normalized conductance	0.05
ω_max_, maximal normalized conductance	1.00

### Network model

In order to employ the above presented neuron model for spike generation in bidirectional all-to-all connecting auto-associative networks, a Hopfield-like network has been implemented^27^. In Hopfield networks all neurons are bidirectionally linked, i.e. *ω*_*ji*_ are the synaptic weights between neuron *i* and *j*, and *ω*_*ij*_ vice versa. Thus, the presented plasticity model has to be extended to account for the bidirectionality of the network. This can be done as follows: The neuron *i* is firing at *k*-th time whenever *u*_*i*_(*t*) reaches the threshold voltage *ϴ*_*thres*_:

6 $${t}_{i}^{k}=t\,if\,{u}_{i}(t)\ge {\theta }_{thres}$$

Afterwards, *u*_*i*_(*t*) is set to the resting potential *u*_*rest*_, while the connections of the *k*-th time spiking neuron *i* (in this case the pre-neuron) to the respective other network neurons *j* (in this case the post-neurons) are set by their actual membrane potential

7 $${V}_{post}({t}_{i}^{k})={{u}_{j}(t)|}_{t={t}_{i}^{k}}.$$

**Figure 3:**
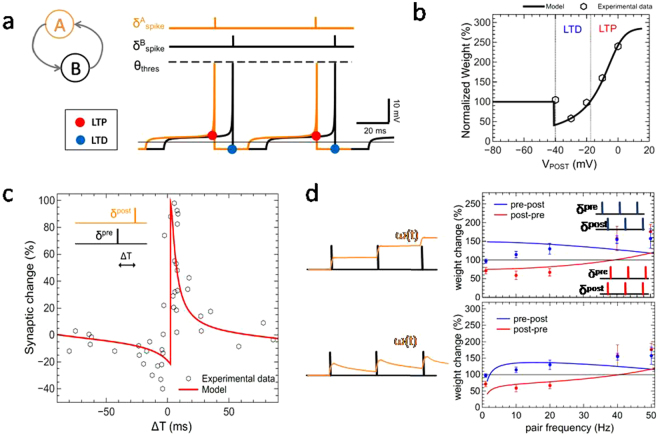
Illustration of the present memristive neural plasticity model in comparison to synaptic plasticity data from neurophysiologic experiments: (**a**) Schematic of the given model: LTP occurs whenever one of the neurons (A or B) is spiking (whichever reaches the threshold voltage *ϴ*_*thres*_) and the membrane potential *u*(*t*) of the opposite neuron is positive (above the black solid line in a). LTD is introduced if at the spike time the opposite neuron has a negative *u*(*t*). Thus, the role of pre and post neuron can change so that a bi-directional coupling between the neurons can be obtained. (**b**) Pre-synaptic stimulation under voltage clamp condition with 25 spike repetitions. Experimental data are taken from ref.^28^ shown as dots. (**c**) Spike-timing-dependent plasticity is compared to experimental data from ref.^2^. (**d**) Frequency dependency of spike pairs for pre-post (blue lines) and post-pre (red lines) pairing. While in the upper graph the synaptic weight is held constant between spike events, a weight dependent depression was applied to obtain the data shown in the lower graph. Solid lines are model data, while dots are experimental data taken from ref.^4^. The used simulation parameters are listed in Table 1. The parameters have been adjusted in such a way that the same set of parameters can be used for all illustrated plasticity emulations.

A schematic with two bidirectionally connected neurons is illustrated in Fig. 3(a). If the membrane voltage of neuron *A* (orange trace) reaches the threshold potential, then their mutual connection is potentiated due to the high positive value of the membrane voltage of neuron *B* (marked by a red points on the black trace). However, if neuron *B* reaches the spiking threshold, the connection to neuron *A* is depressed due to the negative membrane potential of neuron *A* (indicated by the blue dots). Hence, depending on the actual value of the membrane potential of the opposite neuron, either LTD or LTP is induced for their mutual connection.

### Extraction of model parameters from synaptic-plasticity data

The advantage of the proposed neuron model is to limit the number of free parameters. Therewith, the complexity of the model is reduced and a transition to a multi-dimensional network level is feasible. In particular, a very limited number of parameters allow the systematic answer to the question of how each parameter affects the overall network behaviour. The simplicity of the models should nevertheless allow reproducing experimental data in such sense, that the obtained network behaviours contain biological relevance. For the following simulation the model parameters have been chosen to reproduce the experimental data on synaptic plasticity investigations^2–4,28^ and compared to the simulation model of^9^. The used sets of parameters are listed in Tab. 1. We would like to mention, that the presented model further reduces the number of free parameter in comparison to previous published models^9^. Moreover, since the model is also suitable to describe emulation data obtained with memristive devices^19^, it has an additional relevance for the development of real time systems, i.e. neuromorphic systems.

## Results

### Voltage-response functionality for the induction of LTP and LTD

As a first test of the validity of the proposed memristive plasticity model, the synaptic changes as a function of the post-synaptic membrane potential are investigated. Experimental investigation on cells from the hippocampal CA1 slices found that the induction of LTP and LTD strongly depends on the variation of the level of post-synaptic polarization^28,29^. For their experimental investigation, the authors employed the voltage clamp technique, in which the post-synaptic potential is fixed to a reference potential, while the pre-neuron is stimulated.

To emulate such experiments, we set the post-synaptic potential to a fixed value, while for each post-synaptic potential the pre-synaptic neuron input current was employed to evoke a spike train of 25 current pulses. In order to get a direct comparison of the model with the physiological data for the simulation, the used voltage interval has been shifted for the presentation from [−11 mV, 61.75 mV] to [−40 mV, 32.75 mV]. Further, we would like to mention that potentiated synapses are used in the experimental investigation. To account for this in our modeling, the memristive synapses were initialized to *ω* = 0.5, i.e. to the half of their maximal conductance value. The obtained results are shown in Fig. 3(b): the memristive plasticity model is able to reproduce the experimentally recorded voltage dependent data from refs^28,29^, in which LTD and LTP is induced in dependence on the post synaptic potential.

### Spike-timing-dependent plasticity

For a second test of validity of our memristive plasticity model, we reproduced spike-timing dependent mechanisms observed in synaptic plasticity investigations. Therefore, the typical spike-timing dependent plasticity protocol STDP has been reproduced in our model. Here, the pre-neuron is activated shortly before or after the post-neuron is spiking (see sketch in the inset of Fig. 3(c)). Those pairings have been repeated for 60 times at a fixed frequency of 6 Hz. The obtained changes in the weight of the synapse as a function of the relative timing between pre-synaptic spike arrival and post-synaptic firing are compared in Fig. 3(c) with experimental data of Bi and Poo^2^. The comparison shows an excellent accordance of the model data with the STDP experiment.

Additionally, to investigate frequency dependence of the spike pairing, a fixed time delay of +10 ms for (pre-post) or −10 ms (post-pre) was used for pair frequencies ranging from 1 Hz to 60 Hz. The obtained results are compared in Fig. 3(d) to data from cortical pyramidal neurons^4^. For the modeling, two cases has been investigated: while in the first case the weight of the synapse was not affected between the respective spike times, in the second scenario a constant forgetting rate of 4.17 10^−3^ ms^−1^ in ω(t) has been assumed (see sketched in Fig. 3(d)). For both cases, a post-pre pairing (red line) below 40 Hz leads to the induction of LTD, while above LTP is evoked. For the pre-post pairing (blue line) the situation however differs between both cases. Here, the use of a leakage rate in the model (lower panel in Fig. 3(d)) enables us to account for the pair frequency dependent weight change at low frequencies (below 10 Hz), as it was observed in experimental investigations (see blue data points in Fig. 3(d))^4^. In particular, it has been found that pre-post pairings with frequencies below 2 Hz cannot trigger LTP. To account for this in the model, the leakage rate has been added, which provides a constant resetting of the synaptic weight: for low frequencies the spike time initiated weight changes are infrequent, compared to the leakage rate. Therefore, the initiated weight change can be reversed and an even lower value for the weight can be observed, compared to the initial value. At higher pair frequencies, however, the leakage rate is relatively small, compared to the spike repetition rate. Thus, LTP can be triggered (see Fig. 3(d)).

We would like to emphasize, that the assumed leakage rate is realistic for real memristive systems, where a back diffusion of the ions within the memristive layer leads to a loss of memory^23^.

### Connectivity pattern in all-to-all network architectures

**Figure 4:**
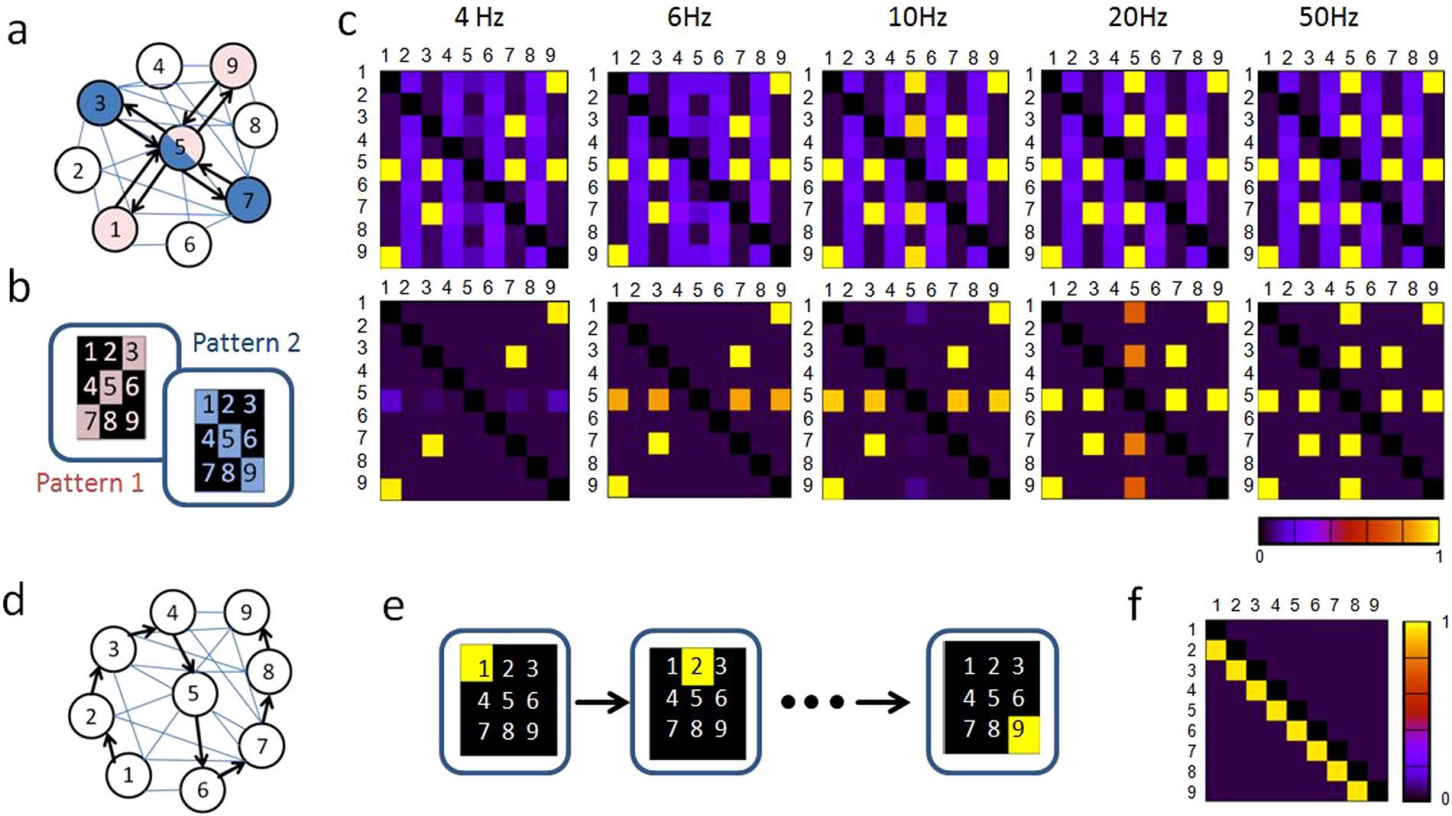
Connectivity pattern in all-to-all connecting networks: (**a**) Schematic of the network topology in which the neurons (1–9) mutually coupled to each other. The colored neurons represent the two applied pattern 1 and 2, shown in (**b**). (**c**) Obtained connectivity patterns (adjacency matrices show the strength of the connection of two neurons each, with rows denoting the jth and columns the ith neurons) for five different spiking frequencies by using rate coding. For the different frequencies the input current to the spiking neurons has been varied. In the upper row the synaptic weight is held constant between spike events, while a weight dependent depression is used in for the simulations shown in the lower row. (**d**) Schematic of the network topology for temporal coding: the black arrows highlighted the connectivity pattern, which are expected to appear for a temporal coding. (**e**) All nine pixels of the input pattern are given successively every 17 ms to the network at a frequency of 60 Hz. (**f**) The weight matrix (adjacency matrix as explained in caption of Fig. 1 (c)) under temporal coding shows the eight unidirectional connections which has been formed in respect to order of showed pixels. For the simulation a constant leakage rate for the single connections has been used. Simulation parameters are given in Table 1, while further technical details of the simulation can be found in the appendix.

In order to investigate the frequency dependent formation of bi-directional connections in a network environment, a system consisting of nine QIF neurons is used (cf. Fig. 4(a)). These neurons are linked to each other in an all-to-all connecting network structure. However, in contrast to Hopfield network structures^27^, the implemented network is bidirectionally linked by two different pathways and not necessarily symmetric. In particular, this allows the investigating of the neural requirements in forming symmetric network topologies and to what extent the plasticity model led to connectivity patterns that reflect the neural code, as it was observed in advanced plasticity models^9^. For the performance investigation, two test patterns are presented to the network, as depicted in Fig. 4b. The obtained connectivity matrixes (weight matrixes) for five different frequencies are shown in Fig. 4c. Therein, two different cases are compared. While for the data shown in the first row, perfect conditions are assumed, namely a complete retention, in the second row data is shown, for which a constant leakage rate of 0.42% per second has been set. As a result, for both cases a frequency dependent increase of bidirectional connections was found, while a constant leakage rate of the memristive connections additionally suppresses those weights, which are not part of the learned pattern. This particularly shows, that the rate coded input is mainly responsible for the formation of bi-directional connections, while a linear depression in terms of a constant leakage rate provides a suppression of all those connections not belonging to the trained pattern.

In particular, the dependency on the post-synaptic voltage enables the formation of receptive fields and allows a linkage between coding and connectivity, as only provided by advanced mathematically plasticity models^9^. This enables the change of the weight evolution in an all-to-all connected network in dependence of the input coding. In order to study this aspect in some more detail, all nine pixels of the input pattern are given successively every 17 ms to the network at a frequency of 60 Hz, i.e. temporal coding is employed (cf. Fig. 4(d,e)). The resulting weight matrix is shown in Fig. 4(f). In contrast to the previous rate coded case, in the case of a temporal coding of the input information unidirectional connections are developed. Since the model parameters and simulation conditions are not changed for the temporal coded simulation, we can conclude that our memristive plasticity models accounts for a coding dependent connectivity, similar to the model of ref.^9^.

### Hippocampal network model of mnemonic discrimination

**Figure 5:**
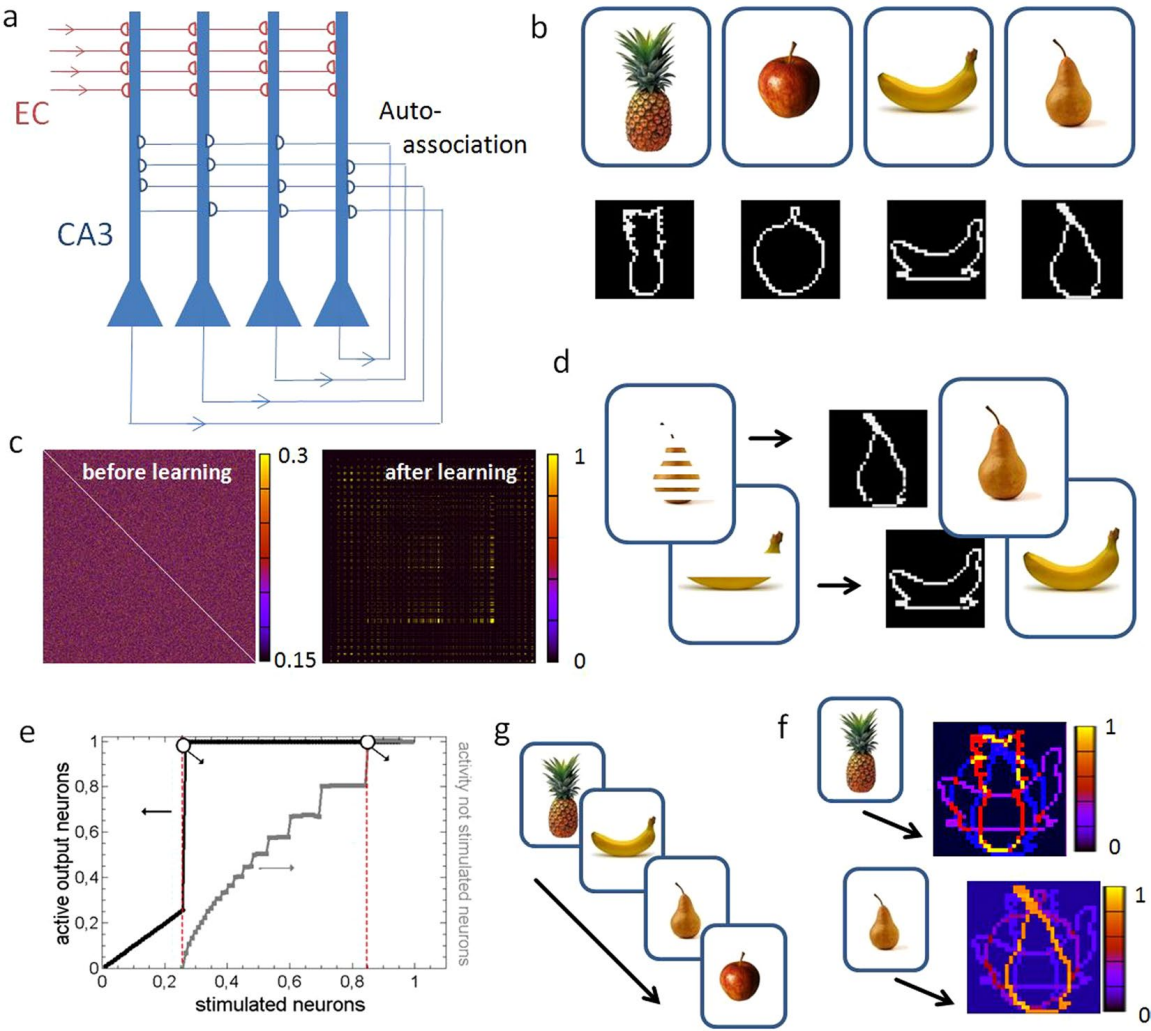
Scalability to the hippocampal circuit:(**a**) Schematic drawing of the CA3 region of the hippocampus, which receive information primarily from the entorhinal cortex (EC). Due to the strong recurrent connections, the CA3 region is assumed to build an auto-associative network with strong bi-directional connections^31–34^. (**b**) MST data used as input pattern to train the network^35^ (the MST behavior task and images are freely available at http://faculty.sites.uci.edu/starklab/mnemonic-similarity-task-mst). For the simulation MST images has been transformed into black and white 32 × 32 pixel images and thereafter their pixel intensities were applied as current values to the model neurons. (**c**) Receptive field of the network, before and after training the network, shows the weights of more than 1 million synaptic connections. Before training, weights are uniformly distributed, after the training matrix of weight shows nearly perfect symmetry (**d**) Incomplete patterns can be completed by the network because of the strong bi-directional connections that have formed during learning. (**e**) Quantitative (gray line) and qualitative (black line) evaluation of completion, depending on the completeness of the original image. **(f)** Schema of temporal coding during training. (**g**) The temporary relationship between the learned sequence and intensity decreases in the output image when the first training image is presented to the trained neural network. Simulation parameters are given in Table 1, while further technical details of the simulation can be found in the appendix.

In order to study the network capability of the implemented memristive model in some more detail, the before described network scheme is applied to emulate the discrimination and processing of mnemonic pattern information within the CA3 field of the hippocampus. It has been shown that hippocampal networks are critical for mnemonic discrimination of similar input patterns (pattern separation). For this purpose, bidirectional connections are required, which results in the formation of local receptive fields to model the auto-associative nature of the CA3 network, as sketched in Fig. 5(a). Beside the recurrent projection within the CA3 network, an external input to the CA3 region is mainly provided from the entorhinal cortex (EC) via the perforant path. For the simulation, an uncrossed unidirectional flow of information has been assumed (cf. 5(a)).

To have a qualitative comparison to behavioral test data, the network was trained with four different images taken from the Mnemonic Similarity Task (MST). The MST is an established behavioral task which assesses pattern separation and pattern completion in the hippocampus^30–32^ and comprises an encoding and a recall phase. In the encoding phase, a set of daily life objects are presented to a participant, while in the recall phase behavioral pattern separation and completion is tested by displaying previously displayed images, images that are similar to the previously seen images, and new images which the participant had not seen before. To use these images within our simulation environment, an edge detection algorithm has been employed to transform the photographs to contour images (cf. Fig. 5(b)). Furthermore, the contours have been centered to the middle of the image and afterwards applied to the network. The respective weight matrixes before and after the learning phase (i.e. before encoding and during recall phase) are shown in Fig. 5(c). As a result, we obtained a symmetric weight distribution from the initial random distributed weights which shows the successful development of bidirectional connections by itself.

In order to test the performance of the network, i.e. to emulate the recall phase of the behavioral test, slight modifications on the MST images has been made as shown in Fig. 5(d). This should mimic the encoding phase in the MST, in which capability of pattern recognition is investigated by showing the participants the same, as well as similar objects from the encoding phase. In this context, it is important to mention that networks of the Hopfield type are not invariant against spatial shift of the input pattern. If the image is shifted by only one pixel, the network will account this as a new image. That is why we used fragmented versions of the original images instead of using pictures of similar objects as it is done in the MST.

The recognition performance of the implemented network is shown in Fig. 5(e). Therefore, the number of applied pixels of the input pattern is varied and the therewith obtained completion of this image has been investigated. As results, we found that an input pattern has to be presented to 27% for the network to complete it. The abrupt jump in the curve underlines the adequate pattern completion performance of the network, as expected for Hopfield networks. Instead of ordinary quotes, a more qualitatively measure of the completion performance of the networks can been analyzed by calculating the quality of the completion

8 $${\rm{Q}}({\rm{e}})=\frac{1}{a{f}_{{\rm{\max }}}}\sum _{n=1}^{a}{f}_{n}(e),$$

where *n* is the running number of internal activated neurons, *a* the number of target pixels, and *f*_*n*_(*e*) the fire rate of the corresponding neuron *n* that changes with the number e of external activated neurons. Further, *f*_max_ defines the maximum fire rate of internal neurons, i.e. *f*_*max*_ measures the maximal internal fire capability of the network neurons due to their auto-associative projections. The obtained results are added as a gray curve into Fig. 5(e). We found that the completion process is already starting when a fraction of 27% of the target pattern is externally stimulated, i.e. only 27% of a prior learned pattern must be presented to the network. However, for a perfect reconstruction of the incomplete input pattern, 85% of the corresponding input neurons must be stimulated externally, whereat perfect is in the meaning of a homogenous completion performance.

Finally, we investigated the network performance for temporal coded (schematic shown in Fig. 4 (d)) input information (Fig. 4 (e)). For this purpose, the four input images are transformed into contour plots and were thereafter sequentially presented to the network, as sketched in Fig. 5(f). In total, 500 loops are used, in which each of these contour plots were applied for 19.2 ms. After the learning phase, i.e. in the recall, the first image (pineapple) is applied to the trained network again. As a result, we found that the three other learned images also appear, but their intensities are reduced in respective to their position in the learned cue. However, if during the recall the third trained pattern (pear) is applied to the network, the last trained picture (apple) is activated with the second highest intensity. Thus, the learned sequence of images can be completely reproduced by the network. This is a particularly important aspect for hippocampal learning and memory formation, in where memory is stored episodically^32–34^, i.e. the hippocampus is known to be the pivotal structure for the formation of episodic memory. Thus, the presented model allows emulating hippocampal key functionalities in a simplified circuit model by using appropriate coding schemes.

## Discussion

Here, evidence has been shown that the presented memristive plasticity model can serve as a basis for the emulation of neural cells of the brain in the context of cellular learning and memory processes, particularly in the hippocampus. For the emulation of learning processes in hippocampal networks, it is important to ensure that new mnemonic information can be stored quickly, flexibly and associatively. In this respect, two processes have a critical function in the establishment of new memory representations: pattern completion and pattern separation^35^. As a structural basis for pattern completion and separation, recurrent collaterals (i.e., the same nerve cell projects onto itself) form auto-association networks^33,34^. These networks consist of separate unidirectional connections, in each case between two neurons, which become bidirectional through the training. The presented memristive model is of particular interest for hippocampal learning, as it allows a connection to the Hebbian learning theory, in which co-actively spiking neurons increase their synaptic coupling weight. This coactivation forms stable activity states (attractor states) by auto-association, as in models described in^32,34^, in which all those activity patterns, which are similar to previous learned patterns, converge towards a stable (learned) attractor state in the absence of further synaptic modifications. Hence, the system facilitates pattern completion. Thus, the synaptic links between those cells are strengthened, which represent different components of the same object and subsequently allow the reactivation of the complete set of original cells by a partial or fragmented subset of cells, that were present during initial learning (cf. Fig. 5). In particular, the formation of bidirectional connections is therefore of importance for pattern completion. This can be addressed with the above described memristive network. Therefore, the memristive plasticity rule enables the formation of bidirectional connections in dependency on the stimulation frequency and coding conditions (cf. Fig. 3).

It is worth to compare our plasticity model with prior published phenomenological models, such as the model published in refs^8,9^. In accordance with this model, a spike-time-dependent plasticity model has been developed, in which synaptic changes are dependent on the spike arrival of the pre-synaptic neurons and the post-synaptic membrane potential. One of the important key contents of the model of refs^8,9^ is that the postsynaptic membrane potential is filtered with two different time constants. Therewith, the weight update differs between the momentary membrane potential and a low-pass filtered version of it, which takes the voltage history over the recent past into account. In this context, memristive behavior provides an excellent opportunity for the simulation of such weight change dynamics, manifesting in a weight dependent learning rate. Thus, in the memristive plasticity model, the strength of the weight change depends on the current value of the post-synaptic membrane potential, the frequency of pre-synaptic firing, and the current weight state itself. We have shown evidence that this makes our memristive model functional comparable to phenomenological neurobiological models and accounts for a variety of non-linear effects that are observed in STDP experiments, as well.

An advantage of the memristive plasticity model is that the model can also be applied to real memristive devices^19,30^. In this context, we were able to show that the described memristive model can be transferred to different types of memristive devices^19,30^, which differ significantly in their resistance change characteristics. Although the model cannot make any statement about the physical mechanisms underlying the resistance switching process, it allows a simple description of the nonlinear behavior of memristive devices. Thus, the model can be applied to a variety of different memristive devices and therefore accounts for the rich variety of memristive devices. This is of particular relevance to the development of neuromorphic computing, since it supports both the design of appropriate memristive devices and the development of complex neuromorphic networks, which take the specific behaviors of memristive devices into account. Therefore, memristive devices are useful to mimic local synaptic features, but can account for global neural network properties as well.

In conclusion, we present a voltage-based plasticity model, which is based on the voltage and history dependence of memristive devices. We showed that the non-linear and state dependent state change of a memristive device is able to account for a variety of experimental plasticity data on STDP and is principally compatible with phenomenological voltage-based SDTP models. In particular, the presented model enables the formation of bidirectional connections and therewith the formation of local receptive fields in auto-associative networks. This might serve as a functional basis for a description of learning and memory formation in the hippocampus. While the presented model is functionally based on an abstract description of a memristive device, we emphasize that the model is also suitable to account for real device hardware. Therefore, we suggest that the presented model helps to implement real-time capable network models, which are able to cope with the high complexity and parallelism of information computing in the brain.

## Methods

### Simulation details

The model has been implemented into a C-code. The neuron model is solved by the classical Runge Kutta method (RK4), while the forward Euler method has been used for solving the differential equation of the memristive plasticity model.

For the network simulation of the CA3 region of the hippocampal field 1024 neurons are recurrently coupled into a bi-directional 1024 × 1023 auto-associative network. Self-projections are forbidden in the simulation model. The input current vector *I*(*t*) to the neurons of the network depends on both, an external input *I*^*ext*^(*t*) and the recurrent synaptic connections *I*^*int*^(*t*):

9 $$I(t)={I}^{ext}(t)+{I}^{int}(t)$$

The constant external input current to the network is defined as

10 $${I}_{n}^{ext}={I}_{0}^{ext}{P}_{n}.$$

Here, *P*_*n*_ is the particular pixel of the 1024 pixels of the input image, which is either 0 or 1. *I*_0_^*ext*^ is a constant, which has been set to 430.25 mA in the simulation. As input pattern images from the mnemonic Similarity Task (MST) have been used, which is a behavioral task designed to tax pattern separation. The test is freely available at the webpage of Stark Lab^38^ and contains different sets of patterns, which show images of daily live objects. To use those images as input patterns in our simulation model, an edge detection algorithm was applied to the images prior to their application to the network. The algorithm set the pixel value in the compressed image to one, whenever the change in the intensity between two neighboring pixels in the original image is above a threshold. This particularly leads to a black-white image containing of binary pixel intensity.

The internal current, which is provided by the recurrent synaptic connections, is given by

11 $${I}_{j}^{int}={\sum }_{i}{\omega }_{ij}\frac{{I}_{0}^{int}\,\delta (t-{t}_{i}^{spike})}{{\sum }_{j}{\omega }_{ij}},$$

where the ${\omega }_{ij}$ are the elements of the adjacency matrix defining the synaptic coupling strengths and *I*_*0*_^*int*^ is the amplitude of the current pulse, which is emitted from a neuron during is spike time *t*^*spike*^. *I*_*0*_^*int*^ has been chosen to 2.4 A in the simulation. Further, the current is normalized by the sum of all connections for convergence.

The network simulation was divided into two phases: training and recall. For training, a time of 7.2 s has been simulated, in which every 19.2 ms the training patterns are changed. Depending on the specific task the network has been trained with two or four patterns. During recall, incomplete versions of the before trained patterns are shown to the network. For better statics, those patterns are applied for a long time of 72 s.
